# The Use of Interdental Care Products in Korean Adults Aged 30 Years and Older and Factors Affecting Their Use: 4th to 7th Korean National Health and Nutrition Examination Survey

**DOI:** 10.3390/ijerph19148639

**Published:** 2022-07-15

**Authors:** Su-Jin Han

**Affiliations:** Department of Dental Hygiene, College of Health Science, Gachon University, 191 Hambakmoero, Yeonsu-gu, Incheon 21936, Korea; sjhan@gachon.ac.kr; Tel.: +82-32-820-4373

**Keywords:** dental floss, interdental brush, interdental cleaning, periodontal status

## Abstract

This study aimed to review the use of interdental care products (ICPs) among adults in Korea, reconfirm their relevance to periodontal health, and identify factors affecting the use of ICPs. Data from 2007 to 2018 from the National Health Nutrition Survey (KNHANES) were used, and 43,069 adults (18,412 men, 24,657 women) aged 30 years or older were included. The frequency and percentage of ICP use according to the characteristics of the subjects are presented. In addition, multivariate logistic regression analysis identified the factors affecting ICP use. The ICP usage rate of participants in the KNHANES phase gradually increased to 22.8% in the 4th, 26.4% in the 5th, and 38.0% in the 6th phase and then decreased to 36.3% in the 7th phase. The adjusted OR values for periodontal health in ICP users were 0.721 for gingivitis and 0.642 for periodontitis, confirming that ICP was associated with a lower prevalence of these conditions. Sex, age, educational level, household income, toothbrushing, and dental check-ups were related to ICP use in all phases. ICP was associated with improved periodontal health, but its use rate was very low. Therefore, oral health professionals should educate adults on the use of ICP, particularly interdental brushing.

## 1. Introduction

According to the Global Burden of Disease 2015 study [1], oral health has not improved over the past 25 years, and as the cumulative burden of oral diseases has rapidly increased owing to demographic changes, including population growth and aging, oral disease is a major public health problem worldwide. Similarly, in South Korea, the total amount of health care insurance benefits due to gingivitis and periodontal disease ranked first in both 2019 and 2020 [2], and the massive economic burden caused by periodontal disease was confirmed.

Periodontal diseases are chronic inflammatory plaque biofilm-related conditions which affect tooth-support structures, leading to tooth loss, and may contribute to systemic inflammation [3,4,5]. Maintaining healthy oral hygiene by effectively removing bacterial plaque from the tooth surface is fundamentally important for the prevention of periodontal disease [4]. Since the effect of reducing the dental biofilm through toothbrushing is only 42% [6], many researchers recommend using an interdental care product (ICP) such as dental floss (DF) or interdental brush (IDB) to clean the proximal space once a day in addition to toothbrushing [7,8]. Direct evidence for an association between interdental cleaning and low periodontal disease in the adult population is still considered weak [9,10]. However, researchers [11] argue that weak evidence should not be construed as ineffective.

According to a meta-analysis on the relationship between the use of ICPs such as DF or IDB and oral health, the use of DF or IDB can contribute to the reduction in gingivitis and dental biofilm compared with brushing alone [12,13]. A recent study reported that toothbrushing and proximal cleaning increase oral health benefits by reducing the risk of periodontitis [14].

The Korea National Health Nutrition Survey (KNHANES), conducted every year in Korea, surveys the frequency of brushing and whether oral care products are used but does not report the results on the usage rate of each product. A previous study [15] reported the changes in oral care product use in 2000, 2003, and 2006 using this data. The use of oral care products by Korean adults confirms that the rate of ICP use is very low, unlike the general practice of brushing teeth more than twice a day. However, after that, only fragmentary data has been reported [16], and no reports had been published on the status of this issue until recently.

Therefore, in this study, the usage of ICPs among Korean adults in a period of 12 years, from 2007 to 2018, was investigated using data from the KNHANES. After confirming the relationship between the use of ICPs and periodontal health, we attempted to identify the factors affecting the use of each product. Through this study, we intend to provide the basic data necessary to recommend and disseminate the use of ICPs to maintain and promote periodontal health.

## 2. Materials and Methods

### 2.1. Study Design

This study used data collected from the 4th to 7th KNHANES (2007–2018) [17,18,19,20]. Administered by the Korea Centers for Disease Control and Prevention (KCDC), the KNHANES is a cross-sectional survey conducted, targeting civilians over the age of 1, and was conducted every three years from the 1st (1998) to 3rd (2005) period. Since then, it has been reorganised into a year-round survey system and has been conducted every year from the 4th period (2007–2009) to the present [21]. The KNHANES is composed of three component surveys: a health interview, health examination, and a nutrition survey. Health interviews and examinations were performed by trained medical staff and interviewers at the mobile examination centre [22]. The KNHANES sampling protocol was designed to include complex, stratified, multistage, and probability cluster surveys of representative samples of the non-institutionalised civilian population of Korea. The CDC Research Ethics Review Board approved the KNHANES protocol and all participants provided written informed consent for participation. For this study, 54,804 adults aged 30 years or older were initially selected from the KNHANES 2007–2018 participants (total of 81,324), excluding 2011 unpublished data (*n* = 8518) and data for participants under the age of 30 (*n* = 26,520). Among them, 43,069 people who had all the information on oral health, general health, and health behaviour were the final study subjects.

### 2.2. Assessment of Periodontal Status

The periodontal status of the participants was evaluated using the Community Periodontal Index (CPI), in accordance with the World Health Organization (WHO) guidelines [23]. The CPI codes were classified as follows: 0, normal periodontal tissue; 1, presence of gingival bleeding; 2, presence of calculus; 3, presence of a 4–5 mm pocket; and 4, presence of a ≥6 mm pocket. In this study, periodontal status was classified with a CPI score of 0 as healthy, 1–2 as gingivitis, and 3–4 as periodontitis. According to standardized protocols, oral examinations were performed by trained dentists [21].

### 2.3. Assessment of the Use of ICPs

The use of ICPs was assessed using the validated Korean version of the Oral Health Questionnaire. The main question was, “Please select all products that you use for your oral health except toothpaste and toothbrush.” The questionnaire did not include specific questions about the frequency or duration of ICP use, thus, this factor was classified only according to whether it was used or not. Individuals using one or more DF or IDB were classified as ICP use. In addition, to check the results according to the type of DF and IDB use, each product was divided into use and non-use categories.

### 2.4. Assessment of Confounding Factors

In this study, risk factors for periodontal disease reported in previous studies [24,25] were considered confounders. Sociodemographic factors included sex, age, education, household income, and residence. Age was classified into six groups: 30–39, 40–49, 50–59, 60–69, 70–79, and ≥80 years. Education level was divided into below high school and above college. Household income was classified into four groups: low, middle-low, middle-high, and high. The residential area was classified as rural and urban. The personal health behaviours included in the analysis were smoking status, toothbrushing frequency, and check-up within the last year. The daily frequency of toothbrushing was categorised as one or less, twice, and three or more times. The systematic medical factors included in the analysis were diabetes mellitus, hypercholesterolaemia, hypertension, and obesity. With respect to diabetes mellitus, participants were classified as normal (<100 mg/dL), impaired fasting glucose (100–125 mg/dL), and diabetic (≥126 mg/dL or taking medication or injecting insulin). Hypercholesterolaemia was defined as a total plasma cholesterol level of ≥240 mg/dL or use of medication. Hypertension was classified into three groups: normal (systolic blood pressure (SBP) < 120 mmHg and diastolic blood pressure (DBP) < 80 mmHg), pre-hypertension (120 ≤ SBP < 140 mmHg and 80 ≤ DBP < 90 mmHg), and hypertension (SBP ≥ 140 mmHg or DBP ≥ 90 mmHg or on antihypertensive medication). Obesity was classified into five groups based on the body mass index value according to the guidelines of the Korean Society for the Study of Obesity [26] and the WHO Asia-Pacific Guideline [27] criteria: <18.5 kg/m^2^, 18.5–22.9 kg/m^2^, 23.0–24.9 kg/m^2^, 25.0–29.9 kg/m^2^, and 30.0 kg/m^2^.

### 2.5. Statistical Analysis

Complex sample analysis was conducted using stratification variables, random, cluster, and weights for all analyses. The first multivariable logistic regression analysis was performed to analyse the association between periodontal status and ICP use and was performed by each KNHANES period. The regression model was adjusted for sociodemographic factors (age, sex, household income, education level, and residence area), personal health practice variables (smoking, toothbrushing, and dental check-up), and systematic medical factors (diabetes mellitus, hypercholesterolemia, hypertension, and obesity). Subsequently, multivariate logistic regression analyses were performed for each phase of the KNHANES to identify factors affecting the use of ICP. In addition, the factors affecting flossing and the use of IDB were identified using the 7th KNHANES data. The explanatory variables were socioeconomic and individual health behaviour variables adjusted for systematic medical factor variables. Data analysis was conducted using IBM SPSS ver. 26.0 (IBM Co., Armonk, NY, USA), and statistical significance was determined at α = 0.05.

## 3. Results

### 3.1. Characterisation of the Study Population According to KNHANES Phase

In total, 43,069 Korean adults (18,412 men and 24,657 women) were included in this study. Of these, 25.2% were healthy without periodontal disease, 34.2% had periodontitis (CPI 3–4), and 40.6% had gingivitis (CPI 1–2) (Table 1).

### 3.2. Characteristics of Those Using Floss and/or Interdental Brushes According to the KNHANES Period

The ICP user rate of the subjects increased to 22.8% in the 4th period, 26.4% in the 5th period, and 38.0% in the 6th period and then decreased to 36.3% in the 7th period. The DF user rate was 12.9% in the 4th period, and it gradually increased thereafter; the use rate in the 7th period was 24.9%. The IDB user rate was 12.6% in the 4th period and 15.0% in the 5th period and increased to 21.2% in the 6th period but decreased to 18.7% in the 7th period (Table 2).

### 3.3. Association between the Use of ICPs and Periodontal Health Status

Table 3 shows the outcomes of the multivariable logistic regression analysis to confirm the relationship between the use of ICPs and periodontal health status. The adjusted OR values for the periodontal health status of the use of ICPs were found to be significantly lower for gingivitis 0.721 (95% CI: 0.676–0.770) and periodontitis 0.642 (95% CI: 0.597–0.690). As a result of subgroup analysis according to KNHANES stage, it was confirmed that the OR of gingivitis and periodontitis was significantly lower when ICP was used. These results were consistent across in all periods from the 4th to 7th phase.

### 3.4. Factors Associated with the Use of ICPs

Factors related to ICP were identified in each phase of the KNHANES. Table 4 shows the probabilities of using ICP, DF, and IDB among Korean adults in a multivariable logistic regression model using the subjects’ demographic characteristics and personal health practices as explanatory variables. The analysis revealed that all variables entered into the model were related to ICP use. Sex, age, educational background, income, brushing, and dental examination were related to ICP use at all phases from KNHANES 4th to 7th, and the association between residential area (5th, 6th) and smoking (4th, 6th) was partially confirmed. The factors affecting the use of DF and IDB were identified using data from the 7th period of the latest survey. The factors related to the probability of using DF were the same as those identified for ICP, and the factors related to the use of IDB were similar, except for the level of income.

## 4. Discussion

The results of this study support the hypothesis that the use of ICP is beneficial to periodontal health. However, ICP use among Korean adults is very infrequent. Oral health professionals must educate adults by constantly emphasising the necessity and importance of cleaning the interdental space in addition to toothbrushing. It is necessary to emphasise the use of IDB in this process.

Based on data from the KNHANES conducted from 2007 to 2018, we examined the status of ICP use among Korean adults over 30 years of age. Interdental cleaning is usually recommended once a day [4,28] or 2 to 4 times a week [29]. In this study, the ICP usage rate was evaluated only in terms of whether it was used or not, not the frequency of use. However, the rate was very low at 22.8%~38.0%, indicating that Korean adults’ ICP usage rate did not reach the recommended level. In particular, compared to the 68.1% of American adults who have been reported to use ICP [24], levels among Korean adults are only half.

According to the Cochrane Review [8], DF or ICP in addition to toothbrushing may reduce gingivitis, biofilm formation, or both more efficiently than toothbrushing alone. In a cohort study conducted in the United States [30], interdental cleaning was associated with a reduction in self-reported gingivitis, and several epidemiological studies [14,29] have also reported that interdental cleaning and the use of DF and IDB among various ICPs could reduce gingivitis and biofilm. In this study, it was also confirmed that when interdental cleaning was performed using one or more DF or IDB, the gingivitis OR decreased to 0.721 (95% CI: 0.676–0.770) and periodontitis OR decreased to 0.642 (95% CI: 0.597–0.690). These results were similar in all phases of the KNHANES. This result reconfirms the finding of previous studies that the use of ICPs is effective in improving the periodontal health of adults. Therefore, oral health experts should consider strategies to increase the use of ICPs.

The factors related to the use of ICP, including DF and IDB, were sex, age, education level, income, toothbrushing, and dental check-ups. The high probability of ICP use in women and high-income earners was similar to that reported in American adults [14], but the effect of smoking as a risk factor for periodontal health was only confirmed in some phases. In addition, the probability of daily interdental cleaning in American adults was found to be higher in the older age groups than in the group of subjects aged 30–44 years [14]. However, in this study, it was confirmed that the probability of using ICP significantly decreased as the age group increased compared to the age of 30–39 years. This trend was also similar to the results of each analysis of DF and IDB use. The use of ICP is essential for interdental cleaning, in particular for removing food residue that accumulates more when the interdental space is exposed because of gingival recession. Therefore, ageing increases the need for interdental cleaning for oral hygiene. However, the opposite trend was observed in Korean adults. Oral health professionals should recognise the responsibility of emphasising the importance of interdental care for middle-aged and older adults and actively guide them to select and use appropriate products.

Toothbrushing at least twice a day is generally recommended for good oral health [4]. However, the probability of using ICP was 1.394 times higher in subjects who brush three or more times than in those who brush only twice. Lee et al. [11] reported that the interaction effect for the prevention of gingivitis and periodontitis was large in subjects who brush three or more times combining toothbrushing with proximal cleaning. When planning an oral health education program, it is necessary to consider and reflect on these points.

It is not clear which product is more effective, DF or IDB, with respect to the effect of using ICPs; however, interdental brushing is effective in reducing interdental bleeding [31], and the effect of interdental brushing has been reported to be superior to that of DF [12]. In addition, previous reports have indicated that the use of IDB can contribute to alleviating periodontal health inequality [32,33]. In fact, it would be more beneficial for periodontal health to promote IDB rather than DF.

In general, flossing is recommended for narrow interdental areas, and interdental brushing is generally recommended for wide interdental areas. Therefore, an IDB may be more useful for adults aged 30 years or older. Therefore, the fact that less than 20% of adults over the age of 30 use IDB is a matter of serious concern.

This study had several limitations. First, since the KNHANES data used in this study are cross-sectional, it is difficult to prove a temporal causal relationship between the use of ICP and the relationship between periodontal health status and the factors influencing the use of ICP. Second, the KNHANES did not include information on periodic use, as only the experience of using each oral care product was inquired. Third, periodontal status was assessed using CPI. The CPI can overestimate or underestimate the prevalence of periodontitis because of the use of the 10 index teeth and the possibility of pseudo pockets [34]. Nevertheless, using a nationally representative sample, we confirmed the changes in the use of ICPs by Korean adults over the past 12 years, verified the need for increased use of ICP based on their relevance to periodontal health, and reviewed related factors.

## 5. Conclusions

In adults over 30 years of age, ICP use was associated with improved periodontal health, but their use rate was very low. Therefore, oral health professionals should actively recommend and educate adults on the use of ICP, and particularly interdental brushing.

## Figures and Tables

**Table 1:** Characteristics of the study population according to KNHANES phase.

Characteristic	Division	Total	4th (2007–2009)	5th (2010–2012)	6th (2013–2015)	7th (2016–2018)
		(*N* = 43,069)	(*N* = 13,041)	(*N* = 9122)	(*N* = 10,720)	(*N* = 10,186)
Periodontal status	Healthy (CPI 0)	10,095 (25.2)	2147 (17.3)	2197 (24.4)	2812 (26.6)	2939 (30.4)
	Gingivitis (CPI 1–2)	17,739 (40.6)	5694 (43.4)	4240 (47.8)	4108 (39.2)	3697 (35.3)
	Periodontitis (CPI 3–4)	15,235 (34.2)	5200 (39.3)	2685 (27.8)	3800 (34.2)	3550 (34.2)
Sex	Male	18,412 (46.7)	5582 (49.8)	3944 (49.8)	4499 (48.2)	4387 (41.2)
	Female	24,657 (53.3)	7459 (50.2)	5178 (50.2)	6221 (51.8)	5799 (58.8)
Age (years)	30–39	9356 (24.4)	3230 (29.2)	2038 (26.8)	2139 (25.3)	1949 (18.7)
	40–49	9725 (25.9)	3160 (29.3)	1969 (28.0)	2340 (26.6)	2256 (21.6)
	50–59	9528 (23.3)	2634 (21.1)	2061 (23.4)	2531 (24.7)	2302 (23.8)
	60–69	8284 (15.2)	2369 (12.5)	1748 (12.8)	2180 (14.3)	1987 (19.5)
	70–79	5307 (9.3)	1420 (6.7)	1166 (7.8)	1372 (8.1)	1349 (13.2)
	≥80	869 (1.8)	228 (1.2)	140 (1.1)	158 (1.0)	343 (3.3)
Education	≤High school	29,704 (65.5)	9794 (70.9)	6403 (68.2)	7223 (62.7)	6284 (61.9)
	≥College	13,365 (34.5)	3247 (29.1)	2719 (31.8)	3497 (37.3)	3902 (38.1)
Household income	Low	8149 (16.0)	2669 (15.4)	1687 (15.6)	1921 (14.3)	1872 (17.9)
	Middle low	10,825 (24.8)	3251 (24.5)	2364 (27.1)	2718 (24.3)	2492 (24.2)
	Middle high	11,906 (29.1)	3545 (29.4)	2495 (28.7)	2988 (30.2)	2878 (28.2)
	High	12,189 (30.1)	3576 (30.7)	2576 (28.6)	3093 (31.2)	2944 (29.7)
Residence area	Rural	9391 (18.4)	3538 (19.5)	1951 (21.3)	2081 (18.2)	1821 (16.0)
	Urban	33,678 (81.6)	9503 (80.5)	7171 (78.7)	8639 (81.8)	8365 (84.0)
Current smoking	Yes	8257 (21.9)	2737 (25.5)	1816 (25.8)	1933 (22.3)	1771 (16.7)
	No	34,812 (78.1)	10,304 (74.5)	7306 (74.2)	8787 (77.7)	8415 (83.3)
Toothbrushing frequency	≤1/day	5131 (10.9)	1781 (12.3)	1171 (12.6)	1180 (10.4)	999 (9.3)
	2/day	17,777 (40.5)	5691 (43.4)	4061 (44.8)	4119 (37.6)	3906 (38.0)
	≥3/day	20,161 (48.6)	5569 (44.4)	3890 (42.6)	5421 (52.0)	5281 (52.6)
Dental check-up	Yes	13,019 (31.9)	3718 (31.0)	2185 (24.1)	3322 (31.5)	3794 (37.6)
	No	30,049 (68.1)	9323 (69.0)	6937 (75.9)	7397 (68.5)	6392 (62.4)
Diabetes	Normal	28,116 (65.6)	9036 (70.2)	6216 (69.5)	6759 (64.5)	6105 (60.7)
	IFG	10,106 (23.8)	2751 (21.0)	1966 (21.4)	2689 (25.3)	2700 (26.3)
	Diabetes	4847 (10.6)	1254 (8.8)	940 (9.1)	1272 (10.2)	1381 (13.1)
Hypercholesterolemia	Normal	35,568 (82.6)	11,475 (88.9)	7683 (85.5)	8722 (83.5)	7688 (75.3)
	Hypercholesterolemia	7501 (17.4)	1566 (11.1)	1439 (14.5)	1998 (16.5)	2498 (24.7)
Hypertension	Normal	20,951 (51.3)	6686 (54.8)	4133 (49.0)	5324 (54.3)	4808 (47.7)
	Pre-hypertension	7713 (17.7)	2221 (16.1)	1722 (18.8)	1923 (17.5)	1847 (18.2)
	Hypertension	14,405 (31.1)	4134 (29.1)	3267 (32.2)	3473 (28.3)	3531 (34.1)
Obesity, body mass index	<18.5	1355 (3.1)	436 (3.2)	287 (3.1)	320 (3.0)	312 (3.1)
	18.5–22.9	16,242 (37.7)	4913 (37.2)	3515 (37.5)	4060 (38.0)	3754 (37.8)
	23–24.9	10,685 (24.7)	3291 (25.3)	2278 (24.5)	2650 (24.6)	2466 (24.5)
	25–29.9	12,941 (30.0)	3911 (30.4)	2700 (30.6)	3204 (29.7)	3126 (29.7)
	≥30	1846 (4.5)	490 (3.9)	342 (4.3)	486 (4.8)	528 (4.9)
Periodontal status	Healthy	10,095 (25.2)	2147 (17.3)	2197 (24.4)	2812 (26.6)	2939 (30.4)
	Gingivitis	17,739 (40.6)	5694 (43.4)	4240 (47.8)	4108 (39.2)	3697 (35.3)
	Periodontitis	15,235 (34.2)	5200 (39.3)	2685 (27.8)	3800 (34.2)	3550 (34.2)

Data are presented as unweighted number (weighted %). IFG: Impaired fasting glucose.

**Table 2:** Characteristics of dental floss and/or interdental brush users.

Characteristics	Division	Interdental Care Products Users											
		Dental Floss or Interdental Brush				Dental Floss				Interdental Brush			
		KNHANES Phase				KNHANES Phase				KNHANES Phase			
		4th (2007–2009)	5th (2010–2012)	6th (2013–2015)	7th (2016–2018)	4th (2007–2009)	5th (2010–2012)	6th (2013–2015)	7th (2016–2018)	4th (2007–2009)	5th (2010–2012)	6th (2013–2015)	7th (2016–2018)
All		2765	2434	3880	3669	1549	1419	2301	2489	1547	1423	2188	1910
		(22.8)	(26.4)	(38.0)	(36.3)	(12.9)	(15.8)	(22.9)	(24.9)	(12.6)	(15.0)	(21.2)	(18.7)
Sex	Male	1036	841	1317	1313	495	436	683	825	642	527	784	731
		(20.3)	(22.0)	(31.8)	(30.1)	(9.9)	(12.0)	(17.1)	(18.9)	(12.4)	(13.3)	(18.9)	(16.7)
	Female	1729	1593	2563	2356	1054	983	1618	1664	905	896	1404	1179
		(25.3)	(30.9)	(43.8)	(40.7)	(15.8)	(19.5)	(28.3)	(29.1)	(12.9)	(16.6)	(23.4)	(20.2)
Age, year	30–39	1026	690	1104	1124	712	524	770	819	464	274	548	580
		(30.2)	(31.5)	(49.7)	(58.1)	(20.8)	(23.3)	(34.5)	(42.7)	(13.9)	(13.1)	(24.8)	(29.6)
	40–49	854	667	1065	1035	454	422	676	728	498	362	582	553
		(26.0)	(31.2)	(44.5)	(46.4)	(13.9)	(18.9)	(27.9)	(33.3)	(14.9)	(17.8)	(24.5)	(24.6)
	50–59	541	566	886	795	239	256	463	546	358	398	540	382
		(21.0)	(25.8)	(34.1)	(35.2)	(9.2)	(11.9)	(17.4)	(24.5)	(13.9)	(17.7)	(21.0)	(16.7)
	60–69	262	350	583	459	113	154	296	276	171	269	347	235
		(12.0)	(17.5)	(26.5)	(23.5)	(5.0)	(8.0)	(13.1)	(14.2)	(7.9)	(12.7)	(16.0)	(12.0)
	70–79	77	151	223	222	28	58	90	108	54	113	158	136
		(5.8)	(11.6)	(15.4)	(15.8)	(1.9)	(4.5)	(6.7)	(7.7)	(4.3)	(8.7)	(10.7)	(9.7)
	≥80	5	10	19	34	3	5	6	12	2	7	13	24
		(2.4)	(5.0)	(14.3)	(11.8)	(1.3)	(2.6)	(4.2)	(3.2)	(1.1)	(3.3)	(10.1)	(9.3)
Education level	≤High school	1593	1360	2143	1656	800	703	1141	993	946	872	1273	926
		(17.6)	(22.0)	(31.3)	(26.6)	(8.9)	(12.0)	(17.0)	(16.3)	(10.3)	(13.5)	(18.2)	(14.6)
	≥College	1172	1074	1737	2013	749	716	1160	1496	601	551	915	984
		(35.5)	(35.9)	(49.3)	(52.1)	(22.4)	(23.9)	(32.8)	(39.0)	(18.3)	(18.2)	(26.2)	(25.5)
Household income	Low	215	200	377	349	98	95	184	182	124	137	244	208
		(9.7)	(13.2)	(20.7)	(19.4)	(4.4)	(6.7)	(10.4)	(10.2)	(5.6)	(8.7)	(13.4)	(11.5)
	Middle low	549	529	903	828	293	290	505	547	310	308	520	439
		(17.7)	(23.2)	(35.1)	(33.8)	(9.5)	(12.6)	(20.1)	(22.2)	(9.9)	(13.4)	(19.8)	(18.3)
	Middle high	874	788	1224	1147	501	469	734	821	492	452	684	568
		(25.1)	(30.6)	(41.8)	(39.8)	(14.2)	(18.9)	(25.5)	(29.1)	(14.3)	(16.8)	(23.5)	(19.2)
	High	1127	917	1376	1345	657	565	878	939	621	526	740	695
		(31.3)	(32.5)	(44.4)	(45.3)	(18.5)	(20.5)	(28.4)	(32.0)	(16.8)	(18.1)	(23.7)	(23.0)
Residence area	Rural	491	287	587	527	242	152	295	307	295	181	370	297
		(17.5)	(16.8)	(29.3)	(30.0)	(8.5)	(9.1)	(14.6)	(17.9)	(10.6)	(9.9)	(18.7)	(16.6)
	Urban	2274	2147	3293	3142	1307	1267	2006	2182	1252	1242	1818	1613
		(24.1)	(29.0)	(39.9)	(37.5)	(13.9)	(17.5)	(24.8)	(26.2)	(13.1)	(16.4)	(21.8)	(19.1)
Current smoking	Yes	473	362	579	556	253	211	299	325	272	202	364	337
		(18.4)	(21.6)	(31.8)	(32.3)	(9.9)	(12.6)	(16.8)	(19.5)	(10.6)	(12.0)	(19.8)	(19.3)
	No	2292	2072	3301	3113	1296	1208	2002	2164	1275	1221	1824	1573
		(24.3)	(28.1)	(39.8)	(37.1)	(13.9)	(16.8)	(24.7)	(26.0)	(13.3)	(16.0)	(21.6)	(18.6)
Toothbrushing	≤1/day	205	137	278	159	110	68	129	87	114	88	163	92
		(14.5)	(12.1)	(25.3)	(16.3)	(7.7)	(6.9)	(12.1)	(9.5)	(8.2)	(7.0)	(14.5)	(8.8)
	2/day	929	903	1252	1173	508	533	737	779	515	504	690	599
		(17.9)	(22.3)	(31.9)	(30.2)	(10.2)	(13.3)	(19.0)	(20.2)	(9.6)	(12.1)	(17.4)	(15.4)
	≥3/day	1631	1394	2350	2337	931	818	1435	1623	918	831	1335	1219
		(29.8)	(35.0)	(44.9)	(44.3)	(16.9)	(20.9)	(27.9)	(31.0)	(16.8)	(20.4)	(25.3)	(22.9)
Dental check-up	Yes	1103	881	1570	1704	629	513	951	1236	633	548	894	875
		(30.4)	(39.2)	(48.2)	(45.2)	(17.5)	(23.0)	(29.6)	(33.0)	(17.2)	(24.0)	(27.2)	(23.1)
	No	1662	1553	2310	1965	920	906	1350	1253	914	875	1294	1035
		(19.4)	(22.4)	(33.3)	(31.0)	(10.8)	(13.4)	(19.8)	(20.0)	(10.6)	(12.1)	(18.5)	(16.1)
Diabetes	Normal	2108	1803	2676	2491	1220	1096	1674	1794	1158	1016	1468	1232
		(24.7)	(28.2)	(41.1)	(41.0)	(14.2)	(17.4)	(26.1)	(29.8)	(13.6)	(15.4)	(22.3)	(20.1)
	Impaired fasting glucose	497	470	890	845	261	248	475	527	288	295	521	463
		(19.7)	(24.6)	(35.0)	(31.2)	(10.9)	(13.8)	(19.1)	(19.6)	(10.9)	(14.7)	(20.6)	(16.9)
	Diabetes	160	161	314	333	68	75	152	168	101	112	199	215
		(14.6)	(17.6)	(25.6)	(25.1)	(6.4)	(7.7)	(12.5)	(12.9)	(9.2)	(12.2)	(15.7)	(16.1)
Hypercholesterolemia	Normal	2453	2086	3211	2867	1392	1263	1936	1992	1360	1178	1782	1465
		(22.9)	(27.0)	(38.5)	(37.7)	(13.0)	(16.5)	(23.6)	(26.5)	(12.6)	(14.9)	(21.2)	(19.1)
	Hypercholesterolemia	312	348	669	802	157	156	365	497	187	245	406	445
		(22.2)	(23.1)	(35.4)	(32.2)	(11.4)	(11.3)	(19.3)	(20.1)	(12.9)	(15.6)	(21.5)	(17.8)
Hypertension	Normal	1753	1340	2360	2167	1063	862	1510	1577	924	716	1269	1063
		(26.2)	(30.4)	(44.7)	(45.5)	(15.9)	(19.7)	(28.9)	(33.4)	(13.8)	(15.9)	(23.9)	(22.0)
	Pre-hypertension	405	453	624	613	208	249	344	396	238	273	361	335
		(20.4)	(27.0)	(35.1)	(33.5)	(10.5)	(14.8)	(19.4)	(21.8)	(11.7)	(16.3)	(20.2)	(18.4)
	Hypertension	607	641	896	889	278	308	447	516	385	434	558	512
		(17.7)	(20.1)	(26.8)	(25.0)	(8.6)	(10.3)	(13.6)	(14.7)	(10.9)	(12.8)	(16.7)	(14.3)
Obesity, body mass index	<18.5	104	91	130	157	60	66	94	121	58	39	63	68
		(25.3)	(33.1)	(40.8)	(53.8)	(14.6)	(24.9)	(27.7)	(42.0)	(14.4)	(13.6)	(22.0)	(22.0)
	18.5–22.9	1150	1046	1654	1481	692	650	1059	1092	613	576	886	710
		(24.7)	(29.7)	(42.9)	(39.7)	(14.9)	(18.0)	(27.8)	(29.5)	(13.1)	(16.2)	(22.8)	(19.1)
	23–24.9	708	602	919	810	360	317	541	538	424	383	510	399
		(23.5)	(24.9)	(36.5)	(32.8)	(12.0)	(13.8)	(21.8)	(22.0)	(13.9)	(15.6)	(20.0)	(15.7)
	25–29.9	707	622	1018	1024	386	340	525	626	393	384	630	597
		(19.7)	(23.5)	(33.6)	(33.1)	(11.0)	(13.6)	(17.9)	(20.2)	(10.9)	(13.7)	(20.3)	(19.3)
	≥30	96	73	159	197	51	46	82	112	59	41	99	136
		(21.4)	(22.9)	(32.5)	(36.8)	(12.0)	(16.2)	(17.5)	(21.8)	(13.0)	(10.9)	(19.6)	(25.3)
Periodontal status	Healthy	679	843	1292	1321	434	545	859	995	359	474	671	625
		(33.3)	(36.8)	(48.3)	(45.2)	(21.6)	(23.7)	(32.5)	(34.1)	(17.4)	(20.1)	(24.8)	(21.2)
	Gingivitis	1246	1112	1550	1433	752	658	953	972	638	625	843	760
		(23.0)	(25.7)	(39.0)	(39.3)	(14.2)	(15.5)	(24.5)	(26.7)	(11.4)	(13.9)	(21.0)	(21.2)
	Periodontitis	840	479	1038	915	363	216	489	522	550	324	674	525
		(17.9)	(18.7)	(28.8)	(25.3)	(7.6)	(9.2)	(13.6)	(14.9)	(11.9)	(12.3)	(18.7)	(14.0)

Data are presented as unweighted number (weighted %).

**Table 3:** Adjusted associations with the interdental care products use and periodontal status according to KNHANES phase.

Explanatory Variable	Periodontal Status	Total	KNHANES Phase			
			4th (2007–2009)	5th (2010–2012)	6th (2013–2015)	7th (2016–2018)
		OR (95% CI)	OR (95% CI)	OR (95% CI)	OR (95% CI)	OR (95% CI)
Interdental care products use (ref. no)	Gingivitis (CPI 1–2)	0.721 ***	0.701 ***	0.729 ***	0.738 ***	0.806 ***
		0.676–0.770	0.612–0.802	0.627–0.848	0.656–0.831	0.715–0.910
	Periodontitis (CPI 3–4)	0.642 ***	0.674 ***	0.644 ***	0.661 ***	0.684 ***
		0.597–0.690	0.581–0.783	0.538–0.770	0.577–0.756	0.601–0.780

*** *p* < 0.001. Response variable: Periodontal status (ref. healthy). The multivariable logistic regression was adjusted for sociodemographic factor variables (sex, age, level of education, household income, and residence area), personal health practice variables (smoking, toothbrushing, and dental check-up), and clinical variables (diabetes, hypercholesterolemia, hypertension, and obesity). OR: odds ratio; CI: confidence interval.

**Table 4:** Odds ratios and 95% confidence intervals of interdental care products use by demographic characteristics and health behaviours among adults aged 30 years or older according to KNHANES phase (2007–2018) and each product in the 7th period.

	4th (2007–2009)	5th (2010–2012)	6th (2013–2015)	7th (2016–2018)	7th (2016–2018)	
	Use of Interdental Care Products				Use of floss	Use of IDB
	OR (95% CI)	OR (95% CI)	OR (95% CI)	OR (95% CI)	OR (95% CI)	OR (95% CI)
Sex (ref. male)						
Female	1.429 ***	1.615 ***	1.687 ***	1.659 ***	1.699 ***	1.342 ***
	1.276–1.600	1.396–1.870	1.488–1.912	1.466–1.876	1.479–1.951	1.170–1.540
Age group (ref. 30–39)						
40–49	0.889	1.103	0.868 *	0.644 ***	0.717 ***	0.770 **
	0.776–1.017	0.930–1.308	0.758–0.995	0.556–0.746	0.607–0.848	0.658–0.902
50–59	0.781 **	1.022	0.627 **	0.474 ***	0.576 ***	0.507 ***
	0.664–0.919	0.835–1.251	0.539–0.728	0.399–0.563	0.478–0.694	0.419–0.614
60–69	0.492 ***	0.727 *	0.509 ***	0.326 ***	0.373 ***	0.378 ***
	0.407–0.596	0.570–0.927	0.426–0.608	0.269–0.394	0.299–0.465	0.305–0.469
70–79	0.246 ***	0.576 **	0.321 ***	0.246 ***	0.244 ***	0.347 ***
	0.182–0.331	0.422–0.786	0.256–0.402	0.198–0.306	0.181–0.327	0.264–0.456
≥80	0.099 ***	0.242 **	0.313 ***	0.202 ***	0.111 ***	0.380 **
	0.039–0.253	0.108–0.545	0.175–0.561	0.116–0.352	0.057–0.217	0.201–0.718
Education levels (ref. ≤high school)						
≥University or college	1.792 ***	1.432 ***	1.459 ***	1.773 ***	1.321 ***	1.310 **
	1.582–2.030	1.203–1.705	1.299–1.639	1.564–2.009	1.133–1.540	1.123–1.529
Household income (ref. low)						
Middle low	1.427 **	1.389 **	1.451 ***	1.257 *	1.379 **	1.189
	1.150–1.771	1.113–1.735	1.222–1.722	1.036–1.526	1.089–1.746	0.950–1.489
Middle high	1.779 ***	1.765 ***	1.609 ***	1.247 *	1.508 **	1.042
	1.450–2.183	1.388–2.246	1.355–1.912	1.033–1.504	1.192–1.908	0.830–1.307
High	2.063 ***	1.721 ***	1.561 ***	1.373 **	1.451 **	1.233
	1.675–2.541	1.347–2.199	1.298–1.878	1.121–1.681	1.135–1.856	0.983–1.547
Residence area (ref. rural)						
Urban	1.096	1.480 *	1.307 **	1.007	1.163	0.962
	0.936–1.282	1.184–1.850	1.143–1.496	0.865–1.173	0.931–1.453	0.792–1.168
Current smoking (ref. yes)						
No	1.269 **	1.132	1.177 *	1.051	1.189	0.874
	1.089–1.479	0.946–1.355	1.010–1.373	0.900–1.228	0.968–1.461	0.736–1.038
Toothbrushing frequency (ref. 2/day)						
≤1/day	1.058	0.677 **	0.977	0.668 ***	0.694 **	0.668 **
	0.869–1.288	0.529–0.868	0.812–1.175	0.539–0.829	0.528–0.911	0.512–0.873
≥3/day	1.538 ***	1.535 ***	1.343 ***	1.400 ***	1.273 ***	1.404 ***
	1.374–1.721	1.353–1.741	1.218–1.481	1.260–1.555	1.123–1.442	1.238–1.593
Dental check-up (ref. no)						
Yes	1.528 ***	1.895 ***	1.659 ***	1.513 ***	1.585 ***	1.372 ***
	1.366–1.710	1.647–2.181	1.503–1.830	1.359–1.685	1.401–1.792	1.221–1.542

* *p* < 0.05, ** *p* < 0.01, *** *p* < 0.001. Response variable was each flossing (ref. no), interdental brush use (ref. no), and use of interdental care products (ref. no). The multivariable logistic regression model was adjusted to clinical variables (diabetes mellitus, hypercholesterolemia, hypertension, and obesity). KNHANES: Korean National Health and Nutrition Examination Survey; OR: odds ratio; CI: confidence interval.

## Data Availability

Publicly available datasets were analyzed in this study. These data can be found here: https://knhanes.kdca.go.kr/knhanes/sub03/sub03_02_05.do (accessed on 5 November 2021).
